# Unilateral abnormality of initial motor-evoked potential in the upper limb detected during lumbar spine surgery: a case report

**DOI:** 10.1186/s40981-024-00708-1

**Published:** 2024-04-10

**Authors:** Sirima Phoowanakulchai, Hironobu Hayashi, Ayako Oi, Yasuhiro Takeshima, Tsunenori Takatani, Masahiko Kawaguchi

**Affiliations:** 1 Department of Anesthesiology, Faculty of Medicine, Siriraj Hospital, Mahidol University, Bangkok, Thailand; 2 Department of Anesthesiology, Nara Medical University, Kashihara, Nara 634-8522 Japan; 3 Department of Neurosurgery, Nara Medical University, Kashihara, Nara Japan; 4 Division of Central Operation, Nara Medical University Hospital, Kashihara, Nara Japan

**Keywords:** Motor-evoked potential, Somatosensory-evoked potential, Train-of-four test, Prone positioning, Lumber spine surgery, Peripheral nerve injury

## Abstract

**Background:**

We present a case with abnormal findings of initial motor-evoked potential (MEP) in the left upper limb after prone positioning during lumbar spine surgery.

**Case presentation:**

A 71-year-old man with bilateral lower extremity numbness without a history of preexisting motor weakness underwent L3–5 spinal fenestration. Initial MEP monitoring after prone positioning revealed markedly prolonged latency and lower amplitude in the left abductor pollicis brevis (APB). Because the left upper limb somatosensory-evoked potentials had normal values, a position-related impending peripheral nerve injury located between the neck and the forearm was excluded. Postoperative examination revealed that MEP abnormality in the left APB was caused by carpal tunnel syndrome.

**Conclusions:**

Abnormal initial MEP from the upper limb was unexpectedly detected after prone positioning during lumbar spine surgery. The condition was caused by preexisting carpal tunnel syndrome.

## Background

Intraoperative neurophysiological monitoring (IONM) is employed to evaluate the functional integrity of nerves during spine surgery. Position-related impending peripheral nerve injury should be considered when abnormal outcomes of IONM of the upper extremities are unexpectedly detected after prone positioning during surgery [1–5].

We describe a case scheduled for lumber spine surgery in which the initial motor-evoked potential (MEP) from the left abductor pollicis brevis (APB) assessed after prone positioning unexpectedly demonstrated amplitude decay and latency prolongation.

## Case presentation

Written informed consent was obtained from the patient for the publication of this case report and any accompanying images. A 71-year-old man who presented with preoperative bilateral lower extremity numbness but preserved motor strength was subjected to L3–5 spinal fenestration for lumbar spinal stenosis, with MEP and somatosensory-evoked potential (SSEP) monitoring. No significant changes were observed at other spinal levels.

The train-of-four (TOF) test as well as MEP and SSEP monitoring was conducted using a neurophysiological monitoring unit (Neuromaster MEE-1232; Nihon Kohden, Tokyo, Japan). For MEP monitoring, transcranial stimulation with a train-of-five pulse at 500 Hz was applied to C3–C4 (International 10–20 system), with the intensity set at the supramaximal level (approximately 500 V). MEP were recorded from the APB on the upper extremities and from the tibialis anterior, gastrocnemius, and abductor hallucis (AH) on the lower extremities. For SSEP monitoring, stimulation was applied over the median nerves bilaterally 3 cm proximal to the wrist and the posterior tibial nerves bilaterally at the ankle with the intensities of 25 and 45 mA, respectively. To record the SSEP, C3′ and C4′ (2 cm posterior to C3 and C4, respectively) were chosen to evaluate the upper limbs and Cpz for the lower limbs, and Fz was established as a reference electrode. A total of 100–200 stimulation repetitions were averaged to record each SSEP. The TOF test was conducted over the mentioned anatomic structures with SSEP-stimulating electrodes. The pattern for the TOF test was composed of four equal stimuli provided at a frequency of 2 Hz, stimulation time of 0.5 ms, and current of 50 mA. The obtained amplitudes of the APB and AH were analyzed. The TOF ratio (T4/T1) was determined by comparing the magnitude of the fourth response (T4) with that of the first (T1).

**Fig. 1:**
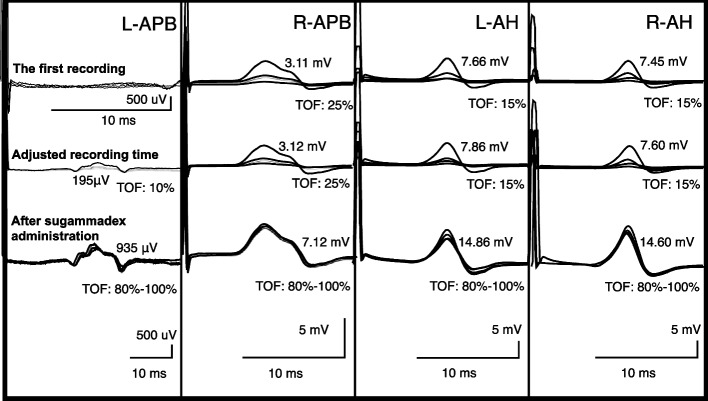
Train-of-four bilateral test conducted at the upper and lower extremities. The first train-of-four (TOF) recordings obtained immediately after prone positioning of the patient exhibited a significantly prolonged latency in the left APB. The recording time was adjusted to observe the left APB waveform (second row). The last row indicates that although the TOF already reached 100%, the left APB had a small amplitude and prolonged latency

**Fig. 2:**
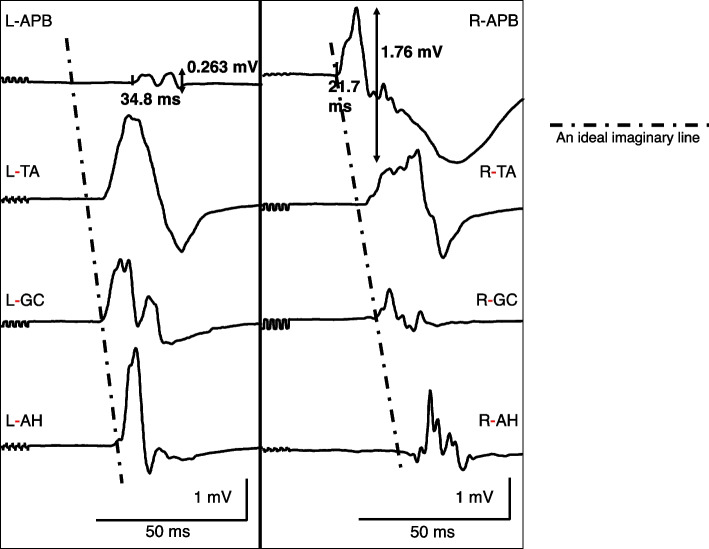
Initial transcranial electrical stimulation motor-evoked potential. The left APB exhibited lower amplitude (0.263 mV) and prolonged latency (34.8 ms), whereas the right APB showed normal values of these parameters (1.76 mV and 21.7 ms, respectively). The ideal imaginary line indicated that the left APB had a significantly prolonged latency

**Fig. 3:**
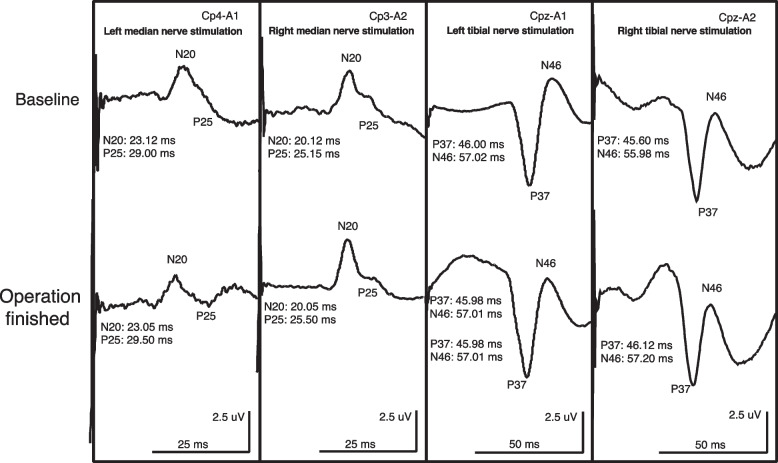
Somatosensory-evoked potentials. Somatosensory-evoked potentials helped identify the cause of the abnormality by demonstrating normal results in all extremities during the procedure

The patient received total intravenous anesthesia using propofol via target-controlled infusion (TCI). Anesthesia was maintained with propofol (2.0–3.0 µg/mL of TCI), remifentanil (0.2–0.5 µg/kg/min), and intermittent bolus injection of fentanyl. The anesthetic depth was regulated to preserve the bispectral index ranging from 40 to 60. No additional neuromuscular blockade was performed to prevent overlapping with MEP waveform interpretations after the administration of rocuronium (0.6 mg/kg) at anesthetic induction. MEP and SSEP were initially recorded after prone positioning. The TOF test showed a 15–25% TOF ratio in the right APB and bilateral AH 45 min after rocuronium administration, while the left APB responses did not appear in the evaluated timespan (Fig. 1). When the analysis window was extended up to three times, the muscle response from the left APB in the TOF test exhibited a TOF ratio of 10% and a markedly delayed latency and a smaller amplitude than those from the right APB. Subsequently, sugammadex (2 mg/kg) was injected to reverse the residual effects of neuromuscular blockade. Although the TOF ratio at all evaluated muscles returned to 100%, the responses from the left APB still exhibited a substantially smaller amplitude than those from the other limbs. Next, the initial MEP was measured, and the left APB demonstrated a substantially prolonged latency (34.8 ms) and a smaller amplitude (0.263 mV) than those of the right APB (21.7 ms and 1.76 mV, respectively) (Fig. 2). Physiological (blood pressure and body temperature), pharmacological (depth of anesthesia), and technical (wiring and equipment settings) parameters and their impact on the MEP were evaluated and found to be appropriate. Among the muscles analyzed, amplitude suppression and latency prolongation were observed only in the left APB; therefore, the systemic influence of pharmacological and physiological factors was ruled out. Because this phenomenon is considered to be a local anomaly, the possibilities of a technical error or impending peripheral neuropathy due to unfavorable body positioning were taken into account. The anesthesiology and neurosurgery teams checked the absence of extreme traction, flexion, and extension of the neck, shoulder, elbow, and forearm, inspected to ensure their neutral positioning. Subsequently, the SSEP from the bilateral upper and lower extremities exhibited normal values (Fig. 3). For the SSEP monitoring at the upper limb, the stimulation point was on the median nerve about 3 cm proximal to the wrist joint. Considering the normal SSEP findings in the upper limb and the neutral position of the neck, shoulder, elbow, and forearm, we ruled out the possibility of a position-related impending peripheral nerve injury originating from the neck and forearm. Finally, peripheral neuropathy at the wrist joint, presumably carpal tunnel syndrome (CTS), was suspected to be the etiological factor for the abnormal TOF and MEP results. As there had been no remedy for this condition, only MEP from the right APB was used as control MEP to assess the systemic effects of the pharmacological and physiological factors. Thereafter, the surgery was completed uneventfully under MEP monitoring, relying on the control MEP from the right APB. Postoperatively, the bilateral numbness in the lower limbs resolved, and there were no new neurological deficits. Postoperative interview with the patient revealed complaints of numbness in the left middle and fourth fingers. Subsequent examination confirmed the diagnosis of CTS.

## Discussion

When abnormal MEP and/or SSEP outcomes from the upper extremity are unexpectedly detected after prone positioning, position-related impending peripheral nerve injury should be ruled out [1–5]. It has been reported that the incidence of position-related SSEP changes in the upper limb during spine surgery is 3.6–6.2% [1–3]. In the present case, we initially suspected position-related impending peripheral nerve injury to the brachial plexus due to the abnormal MEP outcomes unexpectedly detected from the left APB after positional changes. Peripheral nerve injury is the devastating iatrogenic liability in the clinical practice of anesthesiology [6]. Previous studies reported that significant changes in MEP and SSEPs from the upper extremity are the reliable indicators of reversing position-related impending peripheral nerve injuries to brachial plexus during cervical spine surgery [4, 5]. Kamel et al. reported an incidence of 6.2% of position-related upper limb SSEP changes during prone positioning during spine surgery (38 of 609 patients) [1]. Meanwhile, Schwartz et al. reported an incidence of 3.6% of the prevalence of impending brachial plexopathy during surgical correction of scoliosis involving prone positioning according to the outcomes of upper limb SSEP (18 of 500 patients) [2]. Nonetheless, in our case, we ruled out position-related impending peripheral nerve injury to the brachial plexus owing to the normal SSEP findings in the upper limb. We strongly suggested the causative role of the wrist based on abnormalities in the outcomes of the TOF test obtained from the APB with median nerve stimulation. Postoperatively, the patient was eventually diagnosed with CTS.

CTS is the most common focal mononeuropathy due to segmental demyelination, in which the median nerve is compressed along the course in the carpal tunnel. The incidence of clinically diagnosed CTS is 3.8% [7]. Sensory fibers are often affected before motor fibers, and autonomic nerve fibers carried within the median nerve may also become affected. With demyelination, saltatory conduction of action potential is impaired; therefore, the conduction velocity is slowed, and the latency of MEP is prolonged [8]. Reduced MEP amplitude can also occur with demyelination due to secondary axonal loss. Owing to the high frequency of CTS, it may be more rational to target the ulnar nerve rather than the median nerve for the SSEP monitoring and TOF test. Furthermore, the former appears to be more vulnerable, indicating that upper limb SSEP with ulnar nerve stimulation is more effective in detecting irreversible ischemic nerve damages induced by prolonged stretch and contraction of a neurovascular bundle with inappropriate blood flow [3, 9].

The decision of the timing of initial MEP recording, including whether it should be performed before body positioning, relies on several factors. In the case of high-risk spinal instability, where the normal alignment and function of the spinal column cannot be maintained, spinal cord damage following general anesthesia can result from positional changes. In such cases, it may be beneficial to record the initial MEP before positional change [10]. In the present case without spinal instability, MEP were not recorded before the positional change.

During lumbar spine surgeries performed at our institution, we routinely recorded SSEP and MEP in the upper extremity (unaffected side), called the control, to use for identifying truly positive signal change by excluding possible indicators of the systemic effects of anesthetic agents and physiological changes [11]. In our case, the MEP from the left APB was inappropriate as control, and that from the right, APB was only accepted as control. Another option was to record the control MEP from the abductor digiti minimi (ADM), innervated by the ulnar nerve, as no significant differences in the MEP amplitude were observed between the APB and ADM [12].

## Conclusion

This case highlights unilateral abnormality of initial MEP in the upper limb during lumber spine surgery, initially suspected to be position-related nerve injury but later found to be associated with preexisting CTS. Early detection and understanding of underlying conditions are crucial in IONM.

## Data Availability

Please contact the author for data requests.

## Abbreviations

IONM: Intraoperative neurophysiological monitoring
MEP: Motor-evoked potential
SSEP: Somatosensory-evoked potential
APB: Abductor pollicis brevis
TOF: Train of four
AH: Abductor hallucis
TCI: Target-controlled infusion
CTS: Carpal tunnel syndrome
ADM: Abductor digiti minimi
